# Development of a Multiplex Conventional PCR Assay for Concurrent Detection of FAdV-4, FAdV-8b, and FAdV-11

**DOI:** 10.3390/vetsci12020177

**Published:** 2025-02-17

**Authors:** Su-kyung Kang, Dam-Hee Park, Kyeongcheol Min, Sung-Sik Yoo, In-Joong Yoon, Jongseo Mo

**Affiliations:** 1 Choong Ang Vaccine Laboratories Co., Ltd., 1476-37 Yuseong-daero Yuseong-gu, Daejeon 34055, Republic of Korea; sk.kang@cavac.co.kr (S.-k.K.); dh.park@cavac.co.kr (D.-H.P.); minkc220@cavac.co.kr (K.M.); saintyoo@cavac.co.kr (S.-S.Y.); iyoon@cavac.co.kr (I.-J.Y.); 2 College of Pharmacy, Yeungnam University, Gyeongsan-si 38541, Republic of Korea

**Keywords:** fowl adenovirus, polymerase chain reaction, multiplex, poultry, diagnosis

## Abstract

Fowl adenovirus (FAdV) belongs to the *Aviadenovirus* genus within the *Adenoviridae* family. FAdVs are widely distributed and associated with various diseases in poultry, including adenoviral gizzard erosion (AGE), hepatitis-hydropericardium syndrome (HHS), and inclusion body hepatitis (IBH). In this study, we developed a multiplex conventional PCR for simultaneously detecting FAdV-4, -8b, and -11 by targeting the hexon gene. The multiplex PCR was optimized for primer concentrations and thermocycling conditions. The optimal primer concentration combination was set at 0.125 μM for FAdV-4, -8b, and 0.25 μM for FAdV-11. Under these conditions, the limit of detection (LOD) was 10^3^ copies/μL of plasmid standards for FAdV-4, -8b, and -11. These results demonstrated that the developed multiplex PCR method exhibits high specificity and sensitivity, with no observed cross-reactivity among these serotypes or with other poultry viruses. Therefore, this multiplex PCR will be an effective tool for accurate serotyping of FAdV-4, -8b, and -11, enabling more precise identification and differentiation of these three serotypes.

## Simple Summary

A multiplex conventional PCR for simultaneously detecting FAdV-4, -8b, and -11 by targeting the hexon gene was developed. Based on rigorous evaluation, the assay exhibited high specificity and sensitivity, along with no cross-reactivity among the target serotypes.

## 1. Introduction

Fowl adenovirus (FAdV) is a highly contagious, non-enveloped, double-stranded DNA (dsDNA) virus that belongs to the *Aviadenovirus* genus within the *Adenoviridae* family. FAdV is categorized into five distinct species, each distinguished by unique patterns resulting from their restriction fragment length polymorphism (RFLP) profiles [1,2,3]. FAdV is further divided into twelve serotypes based on serum neutralization assays (FAdV-1 to 8a, 8b to 11) [2]. The whole genome of FAdV has a length of 43–45 kb, encoding several structural and nonstructural functional proteins [4]. Among the various types of structural genes, the hexon gene is notable due to its features as subtype-specific antigenic determinants, making it a frequent target for molecular assays for virus detection and routine diagnostics [5]. This is attributed to the hypervariable region in the hexon loop-1 region, allowing the genetic determination of different FAdV serotypes [6]. In addition, the fiber capsid protein, known to initiate the cell binding process, is also involved in determining the virulence and tissue tropism of FAdV [7,8]. FAdV is associated with various diseases in poultry, which include adenoviral gizzard erosion (AGE) [9,10,11,12], hepatitis-hydropericardium syndrome (HHS) [13,14,15], and inclusion body hepatitis (IBH) [16,17,18,19,20]. IBH is characterized by hepatic necrosis associated with intranuclear inclusion bodies in hepatic cells, while typical lesions of HHS include accumulation of yellowish clear transudates in the pericardial sac and necrotic lesions in the liver. Among the various serotypes of FAdV, FAdV-4 from species C is associated with high mortality rates and evident lesions of HHS. AGE can generally be observed in chickens infected with FAdV-1 from species A. The age of infection is another crucial factor, as the severity of the FAdV-associated diseases rapidly increases among younger chicks [21]. Stand-alone infection [22,23] or co-infection with other immunosuppressive viral infections [24,25,26], such as infectious bursal disease virus (IBDV) and chicken infectious anemia virus (CIAV), are both possible in most FAdV field cases. Vertical transmission of FAdV from the parental flock to progenies via embryonated eggs is another critical issue in the industry, making the existence of ‘passed down’ maternal antibodies important [27,28,29,30]. FAdVs are widely distributed worldwide, with different serotypes prevalent in various countries [31,32,33,34,35], causing significant economic impacts on the poultry industry. Thus, consistent surveillance and accurate identification of FAdV serotypes are fundamentally critical. Diagnosis of FAdV infections can be conducted through various methodologies. Among them, polymerase chain reaction (PCR) assay is still one of the most conventional yet popular methods in molecular pathology, using a pair of primers and heat-resistant polymerases to amplify copies of the target DNA [36]. For decades, PCR has been proven useful for detecting etiologic agents of viral diseases. In some studies, PCR was used to either simultaneously detect FAdV alongside other avian viruses with FAdV in a multiplex setting [37,38], or to distinguish FAdV serotypes in a simplex real-time PCR setting [39]. In this study, to aid in the accurate and reliable diagnosis of FAdV, we present a conventional multiplex PCR method for the simultaneous detection of FAdV-4 (Species C), -8b (Species E), and -11 (Species D) that could be conducted on clinical field samples. These serotypes were selected as they were well known as predisposing, emerging causative agents for recent FAdV cases worldwide, particularly in Asia [31,40,41,42,43]. This assay detects the presence of the hexon gene for simultaneous serotyping purposes within each sample. Plasmid DNA standards representing the target sequence of FAdV-4, -8b, and -11 were used to provide authentic standards for hexon gene detection and quantification. Validation and statistical evaluation of the developed assays were rigorously carried out.

## 2. Materials and Methods

### 2.1. Primer Design for Multiplex PCR

Partial or complete hexon sequences were retrieved from NCBI GenBank (https://www.ncbi.nlm.nih.gov/, accessed on 2 May 2023). In this study, forty-four sequences for FAdV-4, forty-four sequences for FAdV-8b, and thirty-five sequences for FAdV-11 were used to identify consensus sequences representing each serotype. The retrieved sequences were aligned using the ClustalW algorithm in MEGA 11 software [44], and consensus sequences were determined for each FAdV serotype. After generating consensus sequences for FAdV-4, -8b, and -11, they were aligned, and the mismatched sequences were used for primer design. The primer designs and sequences are listed in Table 1. The newly designed primers were further validated by an in-depth in silico evaluation using the BLAST search tool at NCBI GenBank.

### 2.2. Determining PCR Sensitivity Using Plasmid DNA Standards

Each genomic DNA of FAdV-4 strain ADL190734, FAdV-8b strain ADL190617, and FAdV-11 strain ADL182047 were used as a template to amplify the target fragments. The PCR products were then purified and cloned into the pTOP TA V2 vector (Enzynomics, Daejeon, Republic of Korea) using TOPO-Cloning based on the manufacturer’s protocols. Each constructed plasmid was transformed into *E. coli* DH5α and cultured in Terrific Broth (Sigma-Aldrich, St. Louis, MO, USA) at 37 °C overnight. The plasmids were extracted using the NucleoBond Xtra Midi Kit (Macherey-Nagel, Düren, Germany), and the concentrations of each plasmid were measured using NanoDropTM 2000 (Thermofisher, Waltham, MA, USA). The plasmid copy number was calculated using the following formula: dsDNA copies = Amount of dsDNA (ng) × Avogadro’s constant (6.022 × 10^23^)/Length of dsDNA (bp) × Conversion factor (1 × 10^9^) × Average mass of 1 bp dsDNA (660). To determine PCR sensitivity, plasmid DNA was serially diluted 10-fold from 1 × 10^10^ copies/μL to 1 × 10^0^ copies/μL.

### 2.3. Optimization of Simplex and Multiplex PCR Assay

This assay used 0.25 U/reaction of *Taq* polymerase (Biofact, Daejeon, Republic of Korea) and 0.2 mM/reaction of each dNTP in fixed amounts. The master mix BioFACT™ 2X Taq PCR Pre-Mix, with Band Helper™ (BioFACT™, Daejeon, Republic of Korea) was used for the multiplex PCR assay. Thermocycling conditions and primer concentrations were calibrated using plasmid DNA standards. Plasmid DNAs mimicking each target serotype were used as templates to optimize the annealing temperature ranging from 56 °C to 64 °C. A mixture of the plasmids from the three serotypes was used to establish the ideal primer concentration. PCR was conducted with the following parameters: pre-denaturation at 98 °C for 5 min, followed by 35 cycles of denaturation at 98 °C for 30 s, annealing at a range from 56 °C to 64 °C for 30 s, extension at 72 °C for 30 s, and a final extension at 72 °C for 10 min. The PCR products were analyzed by electrophoresis on a 2% agarose gel stained with RedSafeTM nucleic acid staining solution (Intron, Seongnam, Republic of Korea). All the plasmid standards and PCR products were validated by Sanger sequencing.

### 2.4. Specificity of the Simplex and Multiplex PCR Assay

To assess the cross-reactivity of each primer set, simplex PCR targeting the three serotypes was performed with Leghorn male hepatoma (LMH) cell-cultured supernatants. Subsequently, to evaluate the specificity of the developed assays, PCR was performed against other poultry viruses, which included avian metapneumovirus (aMPV), avian influenza virus (AIV), chicken infectious anemia virus (CIAV), and infectious bronchitis virus (IBV). Positive PCR results were also added for CIAV, AIV, aMPV, and IBV control viruses.

### 2.5. Sample Preparation for Multiplex PCR

For the diagnosis of FAdV, liver homogenates, and cloacal swab samples are commonly used [45,46,47], while LMH cells and chicken embryo fibroblasts (CEF) are mainly used for FAdV incubation in in vitro settings [34,47]. Therefore, liver homogenates, cloacal swabs, and LMH cell-cultured supernatants were used to determine the efficacy of the newly developed multiplex PCR for clinical applications. The viral strains used in this study included five isolates of FAdV-4, nine of FAdV-8b, and seven FAdV-11, all of which were isolated from poultry farms in Korea. These viruses were kindly provided by Avinext Ltd. (Cheongju, Republic of Korea), a Korean veterinary diagnostic agency. Liver and cloacal swab samples were collected from SPF chickens challenged with each FAdV isolate (FAdV-4 strain ADL190734, FAdV-8b strain ADL190617, and FAdV-11 strain ADL182047). A total of 30 SPF chickens at 9 weeks of age were randomly allocated into three groups (*n* = 10), and each group of SPF chickens was challenged with a corresponding FAdV strain via the intravenous IV route. Challenged birds were monitored for one week, and cloacal swabs were collected daily. In the event of mortality, necropsies were performed at the time of death, and tissue samples were collected during the one-week study. Liver tissues were processed into 10% (*w*/*v*) homogenates in PBS, subjected to three cycles of freezing and thawing, and centrifuged at 8000× *g* for 10 min. Cloacal swabs were treated in 500 μL of PBS and centrifuged at 13,000 rpm for 30 s. The supernatants were collected and preserved at −80 °C. For viral culture, LMH cells were infected with FAdV-4, -8b, and -11 to harvest virus-containing cell-cultured supernatants. Total DNA was extracted from both cloacal swab and cell-cultured supernatants using the MagMAXTM Viral RNA Isolation Kit and the MagMAXTM Express Magnetic Particle Processor (Applied Biosystems, Carlsbad, CA, USA). The extracted DNA was subsequently stored at −20 °C until use.

### 2.6. Verification of Primer Specificity by an In-Depth In Silico PCR

Due to the lack of biological specimens from FAdV serotypes within the same species group as the target serotypes, to further verify the specificity of the multiplex assay and validate the primer designs, a rigorous in silico PCR analysis was performed using FastPCR software version 6.9. Hexon gene sequences from FAdV species C (FAdV-4, -10), species D (FAdV-2, -3, -9, -11), and species E (FAdV-6, -7, -8a, -8b) were extracted in FASTA format from the National Center for Biotechnology Information’s GenBank (www.ncbi.nlm.nih.gov; USA, accessed on 15 February 2024) and processed through FastPCR 6.9 software. This evaluation of the multiplex PCR primer design utilized hexon sequence data from a total of 135 FAdVs (Species C, FAdV-4: 18 strains; FAdV-10: 14 strains; Species D, FAdV-11: 18 strains, FAdV-2: 13 strains, FAdV-3:13 strains, FAdV-9:10 strains; Species E, FAdV-8b: 15 strains, FAdV-8a:16 strains, FAdV-6: 7 strains, FAdV-7: 11 strains).

### 2.7. Ethical Approval

The animal study was approved by the Choong Ang Vaccine Laboratories Institutional Animal Care and Use Committee (IACUC) (permission number 230728-10).

## 3. Results

### 3.1. Primer Design of FAdV-4, FAdV-8b, and FAdV-11

**Figure 1:**
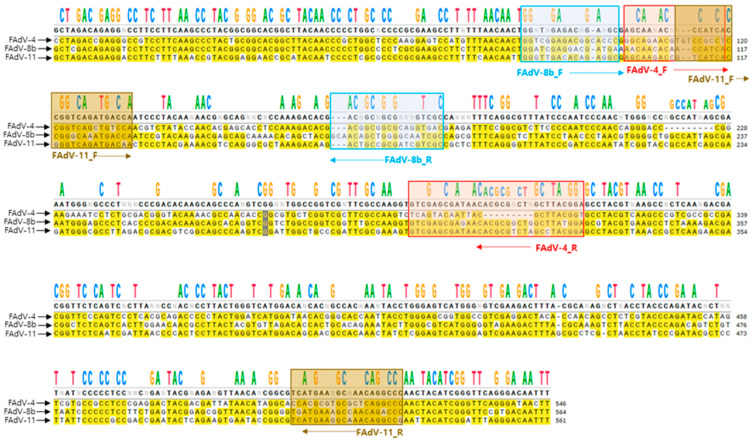
**Primer design of FAdV-4, -8b and -11**. The consensus sequences of FAdV-4, -8b and -11 were aligned, and mismatched sequences were identified. The figure shows the primer binding sites for each FAdV serotype. The PCR amplicon sizes were 212 bp (FAdV-4), 103 bp (FAdV-8b), and 426 bp (FAdV-11), respectively.

Partial or complete hexon sequences of FAdV-4, -8b, and -11 were retrieved from the NCBI Genbank (https://www.ncbi.nlm.nih.gov/, accessed on 2 May 2023) The sequence information is shown in Supplementary Tables S1–S3. The hexon sequences for each serotype were aligned using the ClustalW algorithm in MEGA 11 software. Subsequently, each serotype’s consensus sequences were aligned, and mismatched sequences were identified (Figure 1). Primer-binding sites were selected to generate different amplicon sizes, which were 209 bp for FAdV-4, 103 bp for FAdV-8b, and 426 bp for FAdV-11, respectively.

### 3.2. Optimization of Thermocycling Conditions

**Figure 2:**
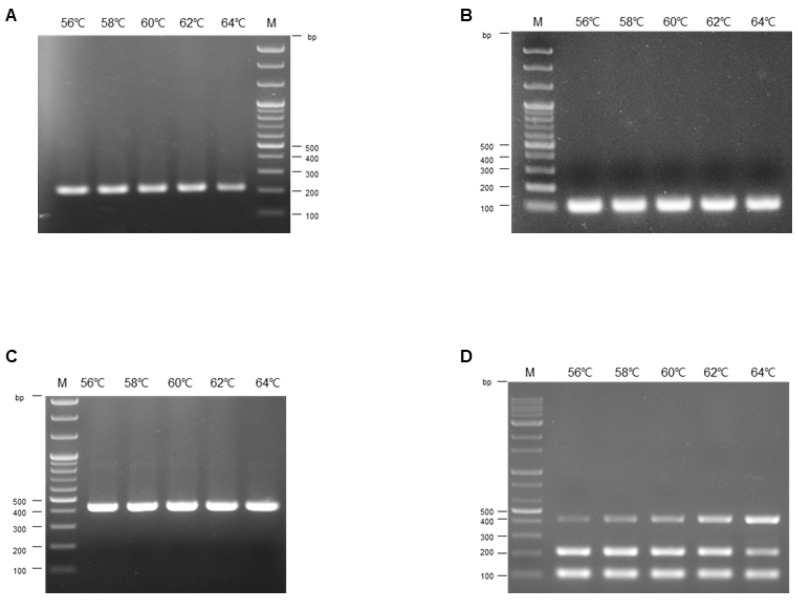
**Optimization of thermocycling conditions**. Simplex or multiplex conventional PCR was performed using single or mixed plasmid DNA standards of FAdV-4, -8b, and -11, with each primer pair set at a final concentration of 1 μM to determine the annealing temperature. (**A**–**C**) Simplex PCR for FAdV-4 (**A**), FAdV-8 b (**B**), and FAdV-11 (**C**). (**D**) Multiplex PCR to determine the optimal annealing temperature.

The optimal annealing temperature for the simplex PCR was initially determined using standard plasmids for each FAdV serotype. The procedure was conducted across a temperature gradient from 56 °C to 64 °C, increasing by increments of 2 °C for each PCR reaction. Each primer set was used at a final concentration of 1 μM. Notably, the simplex PCR results were consistent across the entire range of annealing temperatures from 56 °C to 64 °C, as shown in Figure 2A,C. A combination of standard plasmids for each serotype was used to establish the optimal annealing temperatures for the multiplex PCR assays, each at a concentration of 10^8^ copies/μL per reaction, as shown in Figure 2D. FAdV-11 amplification efficacy increased as the annealing temperature gradually rose, with the final optimal temperature being 64 °C. In contrast, both FAdV-4 and FAdV-8b showed slightly decreased amplification efficiency at 64 °C. At 62 °C, however, all serotypes were appropriately amplified. Therefore, the annealing temperature of 62 °C was determined to be optimal for the multiplex PCR process.

### 3.3. Assessing the Sensitivity of Multiplex PCR Based on Primer Concentrations

**Figure 3:**
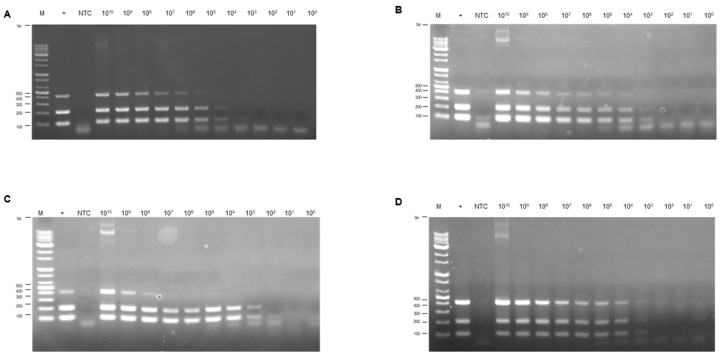
**Assessing the sensitivity of multiplex PCR.** Equal amounts of plasmid DNA standards were mixed and serially diluted, spanning a 10-log range from 10^10^ to 10^0^ copies/μL. The plasmid mixture was added to the PCR reaction. The figure shows the amplification results with different primer concentrations (**A**–**D**). The specific primer concentrations are shown in Table 2; Figure 3A for no. 1, Figure 3B for no. 2, Figure 3C for no. 3, and Figure 3D for no. 4.

The multiplex PCR assay’s sensitivity was evaluated using a balanced mix of standard plasmids representing FAdV-4, -8b, and -11. Different primer concentrations were tested in the multiplex PCR to identify which would yield the highest sensitivity, using four combinations (Table 2). The sensitivity of multiplex PCR was assessed within the range of 10^10^ copies/μL to 10 copies/μL of standard plasmids. The efficacy of the multiplex PCR varied slightly with different primer concentration combinations. At an equal primer concentration of 0.5 μM, the limit of detection (LOD) was 10^5^ copies/μL for FAdV-11 and 10^4^ copies/μL for both FAdV-4 and FAdV-8b, respectively (Figure 3A). Moreover, a decreased primer concentration of 0.125 μM for each set led to reduced sensitivity for FAdV-11, with the LOD dropping to 10^8^ copies/μL (Figure 3C). However, when primer concentrations were standardized to 0.25 μM, or adjusted to 0.125 μM for FAdV-4 and FAdV-8b with 0.25 μM for FAdV-11, the multiplex PCR reached its optimal sensitivity, consistently detecting as few as 10^4^ copies across all three FAdV serotypes (Figure 3B,D).

### 3.4. Assessing the Specificity of Multiplex PCR

**Figure 4:**
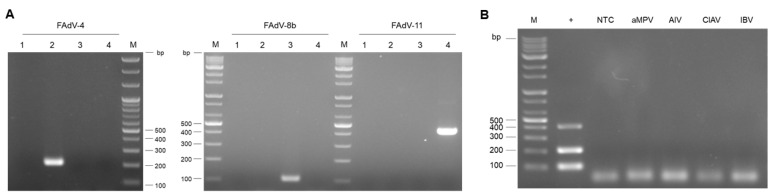
**Assessing the specificity of multiplex PCR**. The specificity of the primers was evaluated through both simplex and multiplex PCR. (**A**) Simplex PCR was conducted using FAdV-4, -8b, and -11 genomic DNA (gDNA) and simplex primer set; Lane 1: Non-template control, Lane 2: FAdV-4 gDNA, Lane 3: FAdV-8b gDNA, Lane 4: FAdV-11 gDNA. (**B**) Genomic DNA from CIAV and complementary DNA from AIV, aMPV, and IBV were used for the specificity analysis of the PCR; +: FAdV plasmid DNA standard mixture served as a positive control. NTC: non-template control.

The specificity of the primers was evaluated through both simplex and multiplex PCR. To confirm the cross-reactivity of PCR for each primer set, simplex PCR for FAdV-4, -8b, and -11 using cell-cultured FAdVs was conducted. The results showed no non-specific amplification or cross-reactivity with the assigned FAdV primer sets (Figure 4A). Additionally, a multiplex PCR analysis was conducted to confirm cross-reactivity for other poultry viruses; aMPV, AIV, CIAV, and IBV. The standard plasmid mixture was used as positive controls. The agarose gel image showed that the three target fragments (FAdV-4, -8b, and -11) were successfully amplified using the standard plasmid mixture. Meanwhile, other poultry viruses were not amplified in the multiplex PCR assay (Figure 4B). Positive control PCR results for CIAV, AIV, aMPV, and IBV control viruses are shown in Supplementary Figure S1.

### 3.5. Evaluating the Multiplex PCR for Clinical Purposes

**Figure 5:**
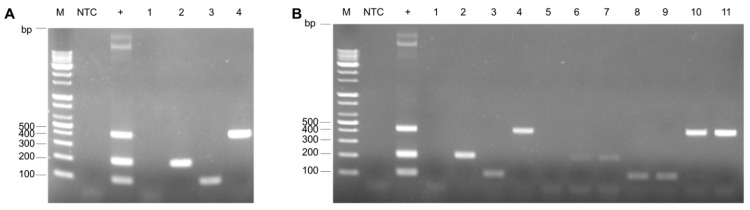
**Evaluating the practicality of multiplex PCR for clinical purposes.** Multiplex PCR was performed with clinical samples and cell-cultured supernatants. NTC: non-template control; +: the positive control. (**A**) Chicken liver homogenates; Lane 1: Not-infected, Lane 2: FAdV-4 infected, Lane 3: FAdV-8b infected, Lane 4: FAdV-11 infected. (**B**) Cell-cultured supernatants (sup) and cloacal swab samples; Lane 1: Mock sup, Lane 2: FAdV-4 infected sup, Lane 3: FAdV-8b infected sup, Lane 4: FAdV-11 infected sup, Lane 5: Not-infected chicken cloacal swab, Lanes 6–7: FAdV-4 infected chicken cloacal swab, Lanes 8–9: FAdV-8b infected chicken cloacal swab, Lanes 10–11: FAdV-11 infected chicken cloacal swab. Multiplex PCR was executed to identify FAdV-4, FAdV-8b, and FAdV-11 strains.

**Figure 6:**
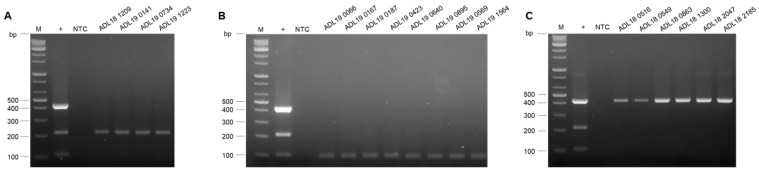
**Assessing the specificity of multiplex PCR**. Multiplex PCR confirmed reactivity with other field FAdV isolates; (**A**) Four strains of FAdV-4 (**B**) Eight strains of FAdV-8b (**C**) Six strains of FAdV-11. Multiplex PCR was executed to identify FAdV-4, FAdV-8b, and FAdV-11 strains. NTC: non-template control; +: the positive control.

In this study, we aimed to develop a practical multiplex PCR that can differentiate FAdV-4, -8b, and -11, for clinical diagnosis. The developed multiplex PCR was evaluated using liver homogenates, cloacal swabs, LMH cell-cultured supernatants, and other field FAdV isolates collected from Korean poultry farms. As a result, the multiplex PCR successfully detected FAdV-4, -8b, and -11 in various sample types without any non-specific amplification (Figure 5). Furthermore, to ensure the specificity of our multiplex PCR, it was tested against various field isolates: four strains of FAdV-4, eight of FAdV-8b, and six of FAdV-11, respectively. The agarose gel results confirmed successfully amplified target-sized bands for each serotype without any non-specific band amplification, as shown in Figure 6.

### 3.6. Primer Specificity Verified by an In-Depth In Silico PCR

In the absence of biological samples for the other serotypes besides FAdV-4, 8b, and 11, computational PCR analysis was carried out using the FastPCR software 6.9, as reported in Supplementary Table S4. The analysis predicted that the tests would effectively produce amplicons from the intended FAdV strains without any unintended cross-reaction with other serotypes within the same species, indicating the high specificity of the multiplex assays.

## 4. Discussion

Since its first report in 1949, FAdV has had a long history of impacting the worldwide poultry industry in the presence of other immunosuppressive diseases or as stand-alone pathogens for decades, causing severe economic implications [40]. FAdV is distributed worldwide, including Europe, Asia, and Africa [33,34,35,47,48]. Considering the primary routes of infection, FAdV is easily transmitted horizontally through the oral-fecal route [49] but is also capable of vertical transmission from the parent bird to their offspring [50]. It is crucial to emphasize that this vertical transmission can occur through embryonated eggs [16], causing high mortality in young chicks. The prevailing understanding of FAdV infection in the past was that it resulted from secondary infections caused by other immunosuppressive infectious diseases, such as IBDV and CIAV. Nonetheless, recent evidence has emerged to support that FAdV can act as stand-alone pathogens, initiating primary infections independently. For instance, It has been proven that FAdV-4 can cause HHS through a single infection [51], while FAdV-8b and -11 have been shown to cause IBH through primary infections [52]. The role of vaccines is critical in the control of FAdV, and vaccination is routinely practiced in commercial poultry operations [31]. Therefore, accurately detecting and differentiating FAdV serotypes within an infected flock is crucial to devising targeted and effective vaccination strategies. For that purpose, we propose a multiplex conventional PCR assay that simultaneously detect and differentiate FAdV-4, -8b, and -11. For stringent verification and standardization, plasmid DNA standards mimicking each serotype’s target sequences (hexon gene) were utilized. Using such plasmid DNA provided rigorous criteria for determining LOD and analytical specificity. Additionally, the assay was further validated with viral DNA extracted from various sample types, including clinical and research samples and other field isolates. After optimization of the primer concentrations, the developed multiplex conventional PCR assay performed equivalently when testing plasmid DNA standards of all three FAdV serotypes, demonstrating linear detection over a 7-log range (10^10^–10^3^ copies/μL) with a LOD of 10^3^ copies/μL for each serotype (Figure 3B,D). Non-specific amplification among these serotypes or other poultry viruses was undetected (Figure 4). All of the known positive samples containing the target serotypes were determined as positive by the multiplex conventional PCR assay, regardless of the sample type (Figure 5). Nevertheless, it’s essential to recognize that solely detecting the hexon loop-1 gene may not always be adequate to provide a definitive diagnosis of a certain serotype. To be more specific, it was observed that the fiber gene, which regulates the infection route of the FAdV, was also genetically variable among different species of FAdV, emphasizing the need to develop a diagnostic assay to detect this specific gene [53]. Consequently, a more sophisticated strategy for FAdV identification that examines not just the hexon gene but also the fiber gene is warranted in the future. It is known that both genes are subject to antigenic variation under immune pressure, making them highly variable [54]. There is also increasing evidence to suggest that FAdVs, particularly species D and E, which include FAdV-11 and 8b targeted in this research, can undergo genetic recombination as a result of co-infections [53]. Such recombination events between different serotypes, which often occur in coinfected cells, could complicate diagnostic outcomes. Coinfections of FAdVs, leading to conditions like IBH or HPS in poultry, have been previously reported [55,56]. Moreover, recently, in China the presence of twelve serotypes from all five species were detected from recent outbreaks [57]

Thus, during outbreaks where multiple serotypes may infect simultaneously, such complexities must be considered when interpreting the results of serotyping tests. However, as recombination typically occurs within the same species group, it is hoped that misinterpretations will be minimal with our currently developed multiplex assay, which targets FAdVs from distinct species groups (FAdV-4, Species C; FAdV-11, Species D; FAdV-8b, Species E). Yet, for future development of multiplex PCR assays aimed at serotyping FAdVs, especially in species groups D and E where recombination is more probable, the inclusion of new target genes like the aforementioned fiber gene or even from the open-reading frames (ORF) in the genome should be genuinely considered [53]

Diagnosis of FAdV can be conducted through various detection methodologies such as gross and histopathological examinations [52], ELISA [50], agarose gel immunodiffusion test, indirect immunofluorescence assay and virus neutralization assay [13], and even transmission electron microscopy [58]. However, these methods are generally time-consuming, laborious and must carefully address potential cross-reactivity issues between different serotypes. Several PCR-based assays have been used to detect and differentiate FAdVs; such as quantitative real-time PCR [39,59,60,61], PCR-based dot blot [62], high-resolution melting-curve analysis [63], LAMP (loop-mediated isothermal amplification) methods [64,65], nanoparticle-assisted PCR assay [66] and digital PCR [67]. Despite their efficiency, these techniques are limited by the high costs associated with experimental materials and the demanding requirements for specialized instruments, which are only sometimes available in diagnostic settings. Besides its high sensitivity, high sequence-specificity, and functional simplicity, the conventional multiplex PCR assay additionally offers an economic advantage by enabling viral gene detection using a smaller sample volume when multiple genetic analyses need to be conducted within the same sample. It also reduces to use of other PCR reagents. Furthermore, it saves time by eliminating the need for multiple PCR runs and reduces pipetting errors since the reaction is carried out within a single tube.

In conclusion, a multiplex conventional PCR assay that can concurrently detect and differentiate FAdV-4, -8b, and -11 in a single tube reaction was successfully developed, allowing this assay to be widely used in research and the field. The provision of this diagnostic assay will facilitate FAdV detection and serotyping in the field

## Figures and Tables

**Table 1:** Primer sequences for FAdV-4, -8b, and -11.

Target Serotype	Sequences (5′ → 3′)		Product Size
FAdV-4	Forward	GGC AGA ACG TGT CCG CCT	209 bp
	Reverse	ACC GTA AGC GTA ATT GTA CTG A	
FAdV-8b	Forward	GGA TCG AGG ACG ATG AAA AC	103 bp
	Reverse	GCG ATT GCC CCA GCT GTT GC	
FAdV-11	Forward	GTC ATC ACG GGT CAG ATG ACA	426 bp
	Reverse	CGG CCT GTT TGC CTT CAT GA	

**Table 2:** Combination of multiplex PCR primer concentrations.

Experimental Number	Primer Concentrations per Reaction		
	FAdV-4	FAdV-8b	FAdV-11
1 (Figure 3A)	0.5 μM	0.5 μM	0.5 μM
2 (Figure 3B)	0.25 μM	0.25 μM	0.25 μM
3 (Figure 3C)	0.125 μM	0.125 μM	0.125 μM
4 (Figure 3D)	0.125 μM	0.125 μM	0.25 μM

## Data Availability

The raw data supporting the conclusions of this article will be made available by the authors on request.

## Supplementary Materials

The following supporting information can be downloaded at: https://www.mdpi.com/article/10.3390/vetsci12020177/s1, Figure S1: Positive PCR results for CIAV, AIV, aMPV, and IBV control viruses; Table S1: Reference of FAdV-4 Hexon gene sequence from NCBI; Table S2: Reference of FAdV-8b Hexon gene sequence from NCBI; Table S3: Reference of FAdV-11 Hexon gene sequence from NCBI; Table S4: In silico PCR prediction of the developed multiplex assay.
