# Supplementary material for: Development of a Multiplex Conventional PCR Assay for Concurrent Detection of FAdV-4, FAdV-8b, and FAdV-11
Source: Vet Sci. 2025 Feb 17;12(2):177. doi: 10.3390/vetsci12020177 (PMC11860461; doi:10.3390/vetsci12020177)
Supplement: Supplementary file 1 [file vetsci-12-00177-s001.zip › Supplementary Figure S1.pdf]

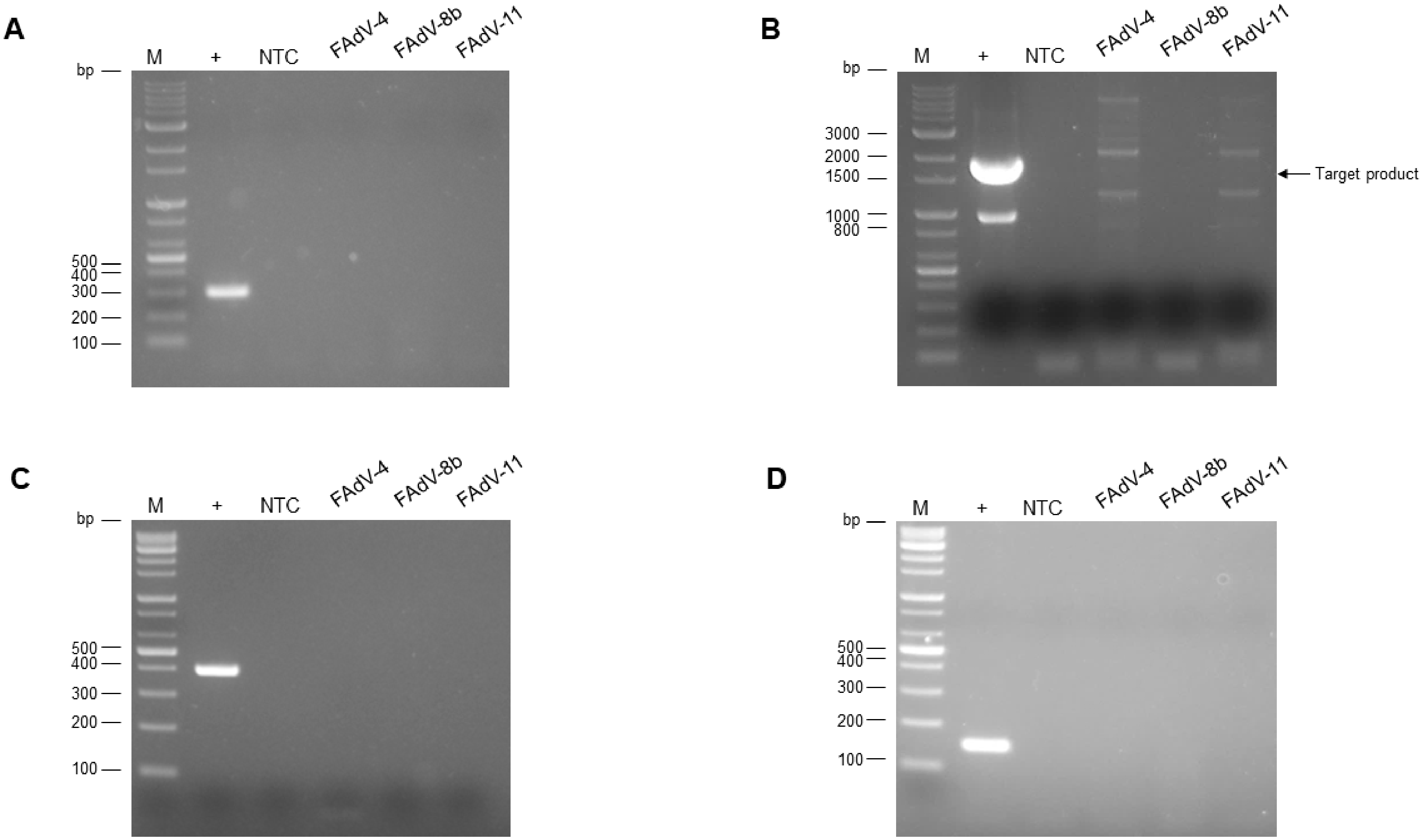

Figure S1. Multiplex PCR analysis was conducted using the designed FAdV-4/8b/11 primers to evaluate cross-reactivity with other common poultry viruses. Each target virus was used as a positive control (+). (A) Avian metapneumovirus (aMPV) (B) Avian influenza virus (AIV) (C) Chicken infectious anemia virus (CIAV) (D) Infectious bronchitis virus (IBV). NTC: Negative control
