# Supplementary material for: Development of a Multiplex Conventional PCR Assay for Concurrent Detection of FAdV-4, FAdV-8b, and FAdV-11
Source: Vet Sci. 2025 Feb 17;12(2):177. doi: 10.3390/vetsci12020177 (PMC11860461; doi:10.3390/vetsci12020177)
Supplement: Supplementary file 1 [file vetsci-12-00177-s001.zip › Supplementary Tables S1-S3.pdf]

Table S1. Reference of FAdV-4 Hexon gene sequence from NCBI

Serotype	Isolate/Strain	Isolated country	Isolated year	NCBI number
FAdV-4	SDJN0105	China	2018	MN102413.1
	CH/CQBS/1609	China	2016	MF055655.1
	CH/SCDY/1606	China	2016	MF055654.1
	CH/SCLJ/1605	China	2016	MF055653.1
	CH/SCDY/1605	China	2016	MF055651.1
	CH/SCYA/1605	China	2016	MF055650.1
	Shandong	China	2015	KT999720.1
	SDSX	China	2015	KT899325.1
	SDJX	China	2015	KT899324.1
	ZK	China	2015	KU647689.1
	YZ	China	2015	KU647688.1
	XZ	China	2015	KU647687.1
	WS	China	2015	KU647686.1
	PDS	China	2015	KU647685.1
	CG-G	China	2015	KU647684.1
	CG-D	China	2015	KU647683.1
	YF1	China	2016	MK650201.1
	QY6-2017	China	2017	MK650200.1
	QY5-2016	China	2016	MK650199.1
	QY3-2016	China	2016	MK650198.1
	QY2-2016	China	2016	MK650197.1
	QY1-2016	China	2016	MK650196.1
	HZ19-2017	China	2017	MK650195.1
	HZ18-2017	China	2017	MK650194.1
	HZ17-1027	China	2017	MK650193.1
	HZ16-2017	China	2017	MK650192.1
	HZ15-2017	China	2017	MK650191.1
	SDBZ-15	China	2015	KU877436.1
	Kr-Yeoju	South Korea	2010	HQ709228.1
	Kr-Gunwi	South Korea	2010	HQ709227.1
	kr-Andong	South Korea	2009	HQ709226.1
	Kr-Cangyeong	South Korea	2008	HQ709225.1
	K09-10	South Korea	2009	JX094358.1
	K09-9	South Korea	2009	JX094357.1
	K07-1	South Korea	2007	JX094354.1
	K531/07	South Korea	2007	HQ697593.1
	ADL 13 3019	South Korea	2013	MN737087.1
	ADL 14 1086	South Korea	2014	MN737088.1
	ADL 15 1132	South Korea	2015	MN737089.1
	ADL 17 0586	South Korea	2017	MN737090.1
	ADL 18 1013	South Korea	2018	MN737091.1
	ADL 19 2139	South Korea	2019	MN737092.1
	ADL 16 0725	South Korea	2016	MN737093.1
	ATCC VR-829	_	_	AF339917.1

Table S2. Reference of FAdV-8b Hexon gene sequence from NCBI

Serotype	Isolate/Strain	Isolated country	Isolated year	NCBI number
FAdV-8b	NSW-6/100932	Australia	2012	KT037700.1
	VIC-11/101089-7	Australia	2013	KT037697.1
	USP-BR-420.12	Brazil	2016	KY229185.1
	USP-BR-420.26	Brazil	2016	KY229170.1
	764	Canada	2009	JN112373.1
	QD2016	China	2016	MF577036.1
	SD14-1	China	2014	MF614119.1
	SD16-113	China	2016	KY426987.1
	SD16-116	China	2016	KY426984.1
	SD1356	China	2016	MG712775.1
	HeB20	China	2020	OK188966.1
	ML001	China	2020	MW284417.1
	SD2009	China	2020	OL456208.1
	FJ-1/100842-C	Fiji	2012	KT037699.1
	FJ-3/100843-F	Fiji	2012	KT37696.1
	50944-M/2015 Debrecen	Hungary	2015	MG953221.1
	35789-M/2015 Debrecen	Hungary	2015	MG953214.1
	UPM04217	Malaysia	2004	KU517714.1
	NZ-1/101151-1	New Zealand	2013	KT037698.1
	ADL17 0258	South Korea	2017	MN737053.1
	ADL17 0511	South Korea	2017	MN737054.1
	ADL17 0644	South Korea	2017	MN797070.1
	ADL18 1204	South Korea	2018	MN767068.1
	ADL18 2817	South Korea	2018	MN737057.1
	ADL18 2838	South Korea	2018	MN737073.1
	ADL19 0066	South Korea	2019	MN737071.1
	ADL19 0167	South Korea	2019	MN737072.1
	ADL19 0187	South Korea	2019	MN737063.1
	ADL19 0240	South Korea	2019	MN737060.1
	ADL19 0310	South Korea	2019	MN737066.1
	ADL19 0329	South Korea	2019	MN737058.1
	ADL19 0337	South Korea	2019	MN737067.1
	ADL19 0423	South Korea	2019	MN737055.1
	ADL19 0434	South Korea	2019	MN737061.1
	ADL19 0518	South Korea	2019	MN737062.1
	ADL19 1065	South Korea	2019	MN737064.1
	ADL19 1224	South Korea	2019	MN737065.1
	ADL19 1466	South Korea	2019	MN737059.1
	ADL19 1573	South Korea	2019	MN737056.1
	ADL19 2356	South Korea	2019	MN737069.1
	ADL19 0569	South Korea	2019	MN737085.1
	ADL19 1564	South Korea	2019	MN737084.1
	ADL19 0895	South Korea	2019	MN737083.1
	ADL19 0640	South Korea	2019	MN737082.1

Table S3. Reference of FAdV-11 Hexon gene sequence from NCBI

Serotype	Strain	Isolated country	Isolated year	NCBI number
FAdV-11	FAdV-11/Brazil/2006/USP-01	Brazil	2006	FJ360747.1
	FAdV-11/Brazil/2007/USP-02	Brazil	2007	FJ360748.1
	USP-BR-102.8D	Brazil	2016	KY229181.1
	USP-BR-418.14	Brazil	2016	KY229169.1
	USP-BR-420.17	Brazil	2016	KY229171.1
	USP-BR-420.18	Brazil	2016	KY229172.1
	USP-BR-420.24	Brazil	2016	KY229176.1
	USP-BR-420.26	Brazil	2016	KY229173.1
	USP-BR-420.27	Brazil	2016	KY229174.1
	USP-BR-420.28	Brazil	2016	KY229175.1
	USP-BR-471.14	Brazil	2016	KY229184.1
	USP-BR-475.1	Brazil	2016	KY229178.1
	USP-BR-475.3	Brazil	2016	KY229179.1
	USP-BR-475.4	Brazil	2016	KY229180.1
	USP-BR-475.11	Brazil	2016	KY229182.1
	USP-BR-G21.B	Brazil	2016	KY229183.1
	USP-EC-02	Ecuador	2016	MF161434.1
	7910/2006 Debrecen	Hungary	2006	KC750777.1
	11069/231/2006 Debrecen	Hungary	2006	KC750778.1
	15843/2010 Debrecen	Hungary	2010	KC750789.1
	15688/2010 Debrecen	Hungary	2010	KC750788.1
	6651/2006 Debrecen	Hungary	2006	KC750776.1
	NZ-2/101151-2	New Zealand	2013	KT037701.1
	ADL13 1851	South Korea	2013	MN737052.1
	ADL13 1863	South Korea	2013	MN737049.1
	ADL14 1036	South Korea	2014	MN737051.1
	ADL15 0666	South Korea	2015	MN737046.1
	ADL17 0387	South Korea	2017	MN737047.1
	ADL18 0354	South Korea	2018	MN737050.1
	ADL19 1676	South Korea	2019	MN737048.1
	K09-21	South Korea	2009	JX094359.1
	K1012/08	South Korea	2008	HQ697595.1
	1047	USA	_	DQ323984.1
	C2B	_	_	AF508959.2
	UF71	_	_	EU979378.1
